# Older Public Housing Tenants’ Capabilities for Physical Activity Described Using Walk-Along Interviews in Montreal, Canada

**DOI:** 10.3390/ijerph182111647

**Published:** 2021-11-05

**Authors:** Kadia Saint-Onge, Paquito Bernard, Célia Kingsbury, Janie Houle

**Affiliations:** 1 Department of Psychology, Université du Québec à Montréal, Montréal, QC H3C 3P8, Canada; saint-onge.kadia@courrier.uqam.ca; 2 Research Center, Montreal University Institute of Mental Health, Montréal, QC H1N 3M5, Canada; bernard.paquito@uqam.ca (P.B.); kingsbury.celia@courrier.uqam.ca (C.K.); 3 Department of Physical Activity Sciences, Université du Québec à Montréal, Montréal, QC H2L 2C4, Canada

**Keywords:** physical activity, older adults, capabilities, public housing, walk-along interviews

## Abstract

Older public housing tenants experience various factors associated with physical inactivity and are locally dependent on their environment to support their physical activity. A better understanding of the person-environment fit for physical activity could highlight avenues to improve access to physical activity for this subgroup of the population. The aim of this study was to evaluate older public housing tenants’ capabilities for physical activity in their residential environment using a socioecological approach. We conducted individual semi-structured walk-along interviews with 26 tenants (female = 18, male = 8, mean age = 71.96 years old). Living in housing developments exclusively for adults aged 60 years or over in three neighborhoods in the city of Montreal, Canada. A hybrid thematic analysis produced five capabilities for physical activity: Political, financial, social, physical, and psychological. Themes spanned across ecological levels including individual, public housing, community, and government. Tenant committees appear important to physical activity promotion. Participants called for psychosocial interventions to boost their capability for physical activity as well as greater implication from the housing authority and from government. Results further support a call for intersectoral action to improve access to physical activity for less affluent subgroups of the population such as older public housing tenants.

## 1. Introduction

Physical activity promotion among older adults is a high priority for public health authorities, since it is known to be salubrious at later stages of life [1,2,3,4,5,6,7] and as older adults are the least active age group worldwide [8,9] Moreover, physical activity is known to be unequally accessible across different groups of the population. As is the case for general health disparities, scholars consistently observe a socioeconomic gradient of physical activity showing lower participation rates among certain subgroups of the population [10,11,12].

Older public housing tenants experience significant inequalities in health. They represent a subgroup of the population with multiple socioeconomic characteristics associated with physical inactivity, which include being of older age, representing a minority cultural background, having lower income and education, presenting higher morbidity, being a woman, and living in a single-person household [8,11,13,14,15,16,17,18]. Moreover, older adults living in public housing face greater vulnerability to poor health. Even when compared to adults with equal age and income living on the private market, older public housing tenants show greater indices of physical and mental illness [15,17,19,20,21]. Furthermore, the social inequalities of health they face include physical activity levels below the recommended levels (<150 min of moderate to vigorous physical activity per week. Nevertheless, studies among older public housing tenants are scarce.

Social and environmental promotion strategies are well-suited to improve access to physical activity for older public housing tenants, since they are less likely to engage in physical activity programs than younger and more socioeconomically privileged individuals [22,23,24] and have limited physical and financial resources [13]. Therefore, this study aimed at gaining a better understanding of the rapport between older public housing tenants’ individual capacity to be physically active, as well as the opportunities and influential factors available to them in their residential environment.

Socioecological models propose that an individual’s physical activity is subject to the influence of their own individual characteristics, as well as the influence of the factors, policies, and norms in the immediate living environment (building); the community (close neighborhood); the wider social and physical environment (municipal, provincial, and federal government); and era [8,25,26]. To be valuable to the individuals living in each environment, the interventions, services, policies, and partnerships that promote physical activity need to be tailored to the needs of its residents [27,28,29]. Indeed, universal interventions are known to omit marginalized subgroups of the population such as older and less fortunate individuals [30,31] which include older public housing tenants. Since they are locally dependent, it seems important to focus on the local aspects of the person-environment fit to gain a better understanding of older public housing tenants’ physical activity.

The capability approach operationalizes the transaction and fit between individual capacities and environmental opportunities as capabilities. Describing economists Amartya Sen and Martha Nussbaum’s work on capabilities, Shinn (2015) defined capabilities as “freedoms to engage in valued social activities and roles—what people can do and be given both in their capacities and constraints in their environments” [32] (p. 243). As a result, capabilities are essential to the experience of a satisfying quality of life [32,33,34]. While recognizing the importance of the social and physical environment, the capability approach also recognizes that each person has their own set of capabilities, since each individual’s experience of life and life aspirations are unique [35,36]. Martha Nussbaum suggested a comprehensive list of eleven capabilities (see Table 1 for the list) [34]. Capabilities are a useful way to describe how individual health behaviors such as physical activity span beyond the individual agency, depending on the interaction with the social and environmental contexts. Evaluating the various dynamic relations between individual capacities and environmental contexts can help in identifying the key points of obstruction or facilitation to being physically active [37]. A capability approach is warranted to further develop health promotion efforts to older public housing tenants, since it is aimed at empowering individuals to make the choices they value for their health by focusing on providing “*equal conditions for fulfilment of peoples’ different desired outcomes*” [36] (p. 2).

To our knowledge, only two studies utilized the capability approach to evaluate older adults’ physical activity—neither among older public tenants. Researchers conducted individual interviews with older adults in Germany [36]. The study’s authors noted three key perceptions shared by most of the participants: First, individuals need to adapt to the changing physical capacity; second, they need to be cognizant of the importance of physical activity for health and wellbeing; and third, environmental infrastructure is unsatisfactory to meet older adults’ needs. Another study conducted in Germany investigated older immigrants’ capabilities for physical activity [37]. This study reports that older Turkish and Russian immigrants felt limited in their ability to be active due to the limited physical capacity and lack of knowledge concerning their specific health, as well as social such as organizational rules, cultural norms, and ageist stereotypes. Both of the studies show the importance of social factors in achieving capabilities to be physically active for older adults. The capabilities related to the built environment appeared relevant in the former study, but not for the older immigrants in the latter study. This discrepancy could be due to the fact that capabilities differ from one context to another. More research is needed to gain a better understanding of older adults’ capabilities for physical activity—especially among lower income older adults, who were few in these studies, as well as among older adults living in different geographical contexts.

In sum, it appears that no studies have investigated older public housing tenants’ physical activity using the capability approach. This work is important since older public housing tenants are seldomly represented in health promotion literature [38,39,40] despite being “underserved” [41] and presenting low indices of health [15,42,43]. The capability approach is a promising avenue to gain a better understanding of the health behaviors in older age [44] and is well-suited to socioecological models of health and wellbeing [45].

### Study Objectives

The aim of this study was to explore the fit between older public housing tenants’ capacities for physical activity and the available opportunities to be active in their residential environment according to a socioecological capability approach.

## 2. Materials and Methods

### 2.1. Design

We used a cross-sectional qualitative explorative study design, since it is geared to investigating lesser-known and experiential phenomena [46]. We conducted 26 individual semi-directed walk-along interviews with tenants of public housing in three neighborhoods in the city of Montreal (Canada).

### 2.2. Methodological Approach

This study implemented an ethnographic approach using walk-along interviews. As researchers, we adopted a constructivist approach contending that new knowledge is formed by linking multiple perspectives [47]. In this case, merging the participants’ and the researcher’s perspectives of older public housing tenants’ capabilities for physical activity in their residential environments. This study received ethical approval from the Ethics Committee of the Université du Québec à Montréal (Certificate number 2080 332).

### 2.3. Participants and Settings

Three public housing sites, exclusively for older adults, were chosen for their residential area diversity (a. residential, b. commercial, and c. mixed) and varying socioeconomic statuses. Individuals were eligible to participate if they were (1) tenants of one of the three study sites; (2) able to walk four 10-min sessions, and (3) able to communicate in either French, English or Spanish. They were excluded if they reported having an intellectual, visual or auditory impairment that could significantly impact walking safety and ability. Participant recruitment is reported in detail elsewhere [48]. We conducted purposeful sampling using posters, snowballing (i.e., word of mouth), and verbal presentation of the study at one social gathering for each site. Moreover, we strived to recruit individuals belonging to subgroups of the population that are typically less represented in research on older adults’ physical activity: Men, less active individuals, as well as those with low physical capacity.

In the metropolitan city of Montreal, a public housing program offers 11,002 housing units to adults aged 60 years and older [49] at a fixed rate of 25% of the tenant’s monthly income. Building units for older public housing tenants are equipped with elevators and community rooms. They do not offer health services. A community organizer visits monthly to provide communication between tenants and the public housing authority, a non-profit organization that manages public housing in Montreal. Its mission is to improve the living conditions and to empower individuals living on a low income, which it also does by offering social and community activities in the buildings [49]. For example, it facilitates the creation and maintenance of tenant committees. An active committee requires a president, as well as a treasurer who must attend a training. Active committees are awarded C$24 dollars per apartment unit per year to subsidize activities for the tenants. Tenant committees are meant to empower tenants, to promote social engagement in the building, and to represent their common interests in discussions with the public housing authority.

### 2.4. Materials

To be inclusive of all the forms of physical activity, this study defined the concept broadly as “moving about”. Three senior researchers developed the interview guide based on an environmental psychology approach to the person-environment fit developed to evaluate the residential environments of disenfranchised populations [28]. Questions included: “How do you feel when you move about here?”; “What makes it easy or pleasant to move around here? What makes it difficult or unpleasant?”

### 2.5. Data Collection

In the walk-along interviews, the researcher and participant travel on foot to the environment of study, while discussing it in real time. This allows the gathering of a rich pool of information, since they place the interviewee in the studied context or “in situ” [50]. In addition, it allows the researchers to assess how factors emerging from the physical, social, and individual dimensions interact in their influence on physical activity [51]. Moreover, walk-along interviews create an informal atmosphere anchored in the participant’s daily life, which stimulates informal conversation and sharing [51].

The method allows the simultaneous collection of ethnographic observations of the physical and social environment, as well as the perceptions of the concerned individuals [51]. Based on a constructivist approach, rather than rejecting the subjectivity of the individuals involved, we considered meaningful information to be co-constructed [52] between the participant (informant) and the interviewer (learner), who actively receives information and challenges it using their observation of the environment. As a self-reflective co-producer of knowledge, the interviewing researcher (First author) took the liberty of testing out budding interpretations with participants.

During the initial data collection, the interviewer was dubious of how few facilitators and barriers to physical activity in the residential environment were being mentioned and how resigned to their current living situations participants appeared to be. Participants stated that “everything is good”, while the researcher observed contradictory information such as aspects of the built environment that participants were unable to manoeuvre, yet consistently neglected to mention. During a telling interview the researcher, feeling stuck and frustrated with how improbable it seemed to her that participants did not aspire to any improvements at all compared to those found in the scientific literature on older adults’ physical activity, resolved to reveal her astonishment and use “guiding questions”. Kirkevold and Bergland (2007) noted that this technique may resemble “leading questions” [53] (p. 74), but that, in fact, framing perceptions and interpretations in the form of closed interview questions is a useful strategy to gather greater and richer information from laconic participants. Therefore, to adapt to this study context, we included these conversational interviewing techniques, which are said to promote reflexivity of both the interviewer and interviewee [54].

Reflexivity can be described as (1) directing awareness to oneself in relation to an object of study to consider why and how we are interpreting it, not solely what our interpretation of it is; and (2) being sensitive to how the dynamics of power between the researcher and interviewee may influence the discourse between the two [55]. Moreover, it is an ethnographic tool by which to address the researcher’s subjectivity of the object or population of study. Stimulating reflexivity during an interview not only makes for a more natural, contextually focused conversation, greater reflexivity in both the researcher and participant also promotes analytic depth during the data collection process [55,56]. Furthermore, the interviewer practiced reflexive journaling for 20 to 60 min following each interview on top of taking observational field notes [55]. These measures allowed the interviewer to gain an awareness of how her perceptions may influence the data, as well as to uphold a qualitative analysis rigor [55].

### 2.6. Procedures

The interviewer read the information and consent form with each participant before obtaining written and oral consent to participate. Data collection occurred from 11 September to 25 October 2017 and lasted between 1 and 2.5 h. The interviews were audio recoded and transcribed verbatim.

In the first part of the interview, older public housing tenants described physical activity in their own words during a preliminary sit-down interview (15 min), which also served to build rapport between the interviewer and interviewee. Second, participants described opportunities as well as the factors that help or hinder physical activities in their residential environment by leading the interviewer on foot to a space where they tended to be active in their apartment (10 min), their building (10 min), and their close neighborhood (2 × 10 min). Participants were asked to choose a place in their close neighborhood within 10 min on foot where they typically moved about (e.g., walking to the bank or attending the local YMCA) taking as many breaks as necessary. When possible, the interviewer and participants took alternative routes to gain information on novel stimuli [57]. Finally, participants completed an 18-item homemade questionnaire gathering sociodemographic information such as age, annual household income, country of birth, and perceived health status. Furthermore, participants completed the functional comorbidity index [58], which counts the sum of 18 past or present chronic health conditions, as well as the Physical Activity Questionnaire for the Elderly, which classifies the time spent in physical activities during the last 7 days as either “high”, “moderate” or “low” [59].

### 2.7. Data Analysis

Interview transcripts were uploaded to the qualitative coding software NVivo versions 11 and 12 (QSR International, 2015 and 2017) and evaluated using a five-step approach to thematic analysis [60]. Two researchers (first author, a doctorate student in community psychology and third author, a bachelor student in kinesiology) first got acquainted with the corpus of data, noting initial codes. Second, they performed inductive open coding, cross-coding 10% of the corpus to establish common coding strategies. Third, the interviewer-coder applied a deductive approach of coding and grouping codes into themes of capability. Moreover, this main coder identified the socioecological systems mentioned or implied in the participants’ discourse. Four main systems were found: The individual, the public housing building, the community (including the neighborhood), and all the levels of government. Fourth, the interviewer-coder reviewed the themes by reading transcript excerpts and comparing them to her reflexive journal and observational field notes. Finally, she selected significant excerpts to define each theme.

The research team supported the rigor of qualitative analysis using (1) “member reflections”, (2) “critical friends”, and (3) quality criteria adapted to this study [61]. As described above, member verification occurred using reflexive and conversational interviewing techniques during data collection. Researchers met regularly throughout the data collection and analysis to iteratively challenge their interpretations along with a panel of critical friends including graduate students from diverse sub-disciplines of psychology and kinesiology. Finally, quality criteria were to triangulate the transcript analysis with the main researcher’s reflexive journal and field notes; to search for positive examples of capabilities as well as constructive critique; to explore counter examples with which to challenge interpretations; to respect the participants’ spoken word but also their anonymity; and to produce information of practical use for decision makers and health promoters.

## 3. Results

In general, similar to older public housing buildings in Montreal, our sample comprises more women (69%) than men (31%) and slightly more Canadian-born tenants (54%) than not (46%). Participants were aged between 60 and 93 years old. Two thirds of the sample reported low physical activity levels over the past week, while the other third reported a moderate level. Table 2 details the sample’s sociodemographic and health characteristics. In what follows, we describe five capabilities for physical activity: Political, financial, social, physical, and psychological. Moreover, though participants displayed a positive outlook, the impetus for this paper was a striking underlying mood of resignation in the participants’ discourse.

### 3.1. Political Capabilities

Political capabilities are the fit between tenants’ capacity to exert change onto their residential environment and the opportunities to be physically active within it. Participants evoked political capability especially when discussing the tenant committee, the public housing authority’s regulations, as well as multiple levels of government.

Tenant committees were said to be paramount to accessing physical activity in all of the three study sites, although only one site had an active committee. Its recently appointed president was organizing physical activities for the tenants, seeking to renew a previous partnership with the YMCA to offer low-cost yoga onsite. Participants described committee presidents as leaders, someone to advocate for tenants and to bring them together. For example, a previous president of one site obtained funding directly from the elected municipal deputy to equip the community room with cardiovascular training equipment (treadmills, elliptical machines, and stationary bikes). A participant described the transaction as political power:

“It’s the tenant committee that asked that we receive these machines specifically thanks to people’s power.”

[C030]

Moreover, participants mentioned various public housing authority regulations that impacted their physical activities. For example, dog ownership was recently allowed in one of the study sites. However, participants in all of the three sites described having little to no decision-making power concerning their own residence. For example, participants of one site mentioned that new plans for common spaces were presented during a monthly tenant meeting without the possibility of weighing in on those changes. This, they said, could have been an opportunity to request spaces for physical activity. Participants from all of the study sites mentioned that greater cleanliness in the building would encourage physical activity within it. Nevertheless, they recognized that the public housing authority’s budget was overstretched, and their custodians were overworked. Participants concurred that nothing could or would be done to improve the built environment in the building. When the interviewer asked what would happen if the disjointed walkways around one building’s yard were joined, one participant noted a sense of resignation to the current status quo:

B070: Well, that would be easier to access. I realize it would be easier to get there, but I hadn’t stopped and thought about it until you brought it up. Since I don’t often do that, I didn’t realize: “Ah, shucks, it’s too bad I can’t go through there.” Well, no, I’d have to lift [the walker] up here, but since I don’t do it often, I didn’t feel the need…

Q: But in fact, you mentioned a little earlier not realizing it. That’s what I keep hearing from most people: “we don’t realize it”…

B070: We take for granted; well, we don’t take for granted but we take it for granted that’s the way it is. We don’t think about how much better it would be for us.

Q: What do you think about that?

B070: Maybe it’s a bad habit…

A few participants who questioned the role that the public housing authority plays, genuinely wondered if its scope of practice is reserved to the building management and, if that is the case, they wondered why that was. At all of the three sites, participants suggested that the public housing authority should play a larger role in the building, one that is closer to the tenants. For example, they wished the public housing authority would also tend to the individual and collective living to improve their wellbeing and health. A common suggestion was for the public housing authority to consult and involve its tenants and draw not only from their lived knowledge, but also their professional knowledge as well as their participation.

“Well, we won’t go as far as to ask why the ministry is making [budget] cuts, that’s too far for us. But let’s come back here to… to [social housing]. We want to solve the problem in [social housing]. We don’t want to create problems. How can we solve this problem? We don’t have staff, we have volunteers. There are people here who are volunteers, we will do the cleaning. If, for example, the management here doesn’t have good counsellors, we have psychotherapists here. Does the management ask them for advice? They could ask them to take [charge]. But if the management it’s not their prerogative, their prerogative is just to make repairs. If a tenant is sick it’s up to that person to manage on their own…”

[A060]

This sense of resignation continued in participants’ discourse of government support. For example, they were very grateful for municipal-funded activities such as Zumba in the park, as well as the federal-funded old-age pension. Nevertheless, they expected little else from the government in terms of age-friendly improvements to the built environment such as better-quality sidewalks or stricter, safer speed zoning. A common understanding among the participants was either that you should be grateful for what you have or that you cannot argue with those in power:

B090: No, no. You cannot. You’d be talking in a vacuum.

Q: All the time?

B090: That’s it. So, you can’t be against authority.

Q: Aren’t the city people there to make things better for the residents?

B090: They’re the ones who manage the social housing, they’re the ones who run the city. They have all the power.

Q: How do they use their power? For the good…

B090: Well, it’s the budget! It not any more complicated than that. It’s about what pays for itself.

Q: What do you think they should do?

B090: They do what they have to do to make it profitable.

Q: Because it’s the right way to do it?

B090: No, they don’t go according to the people.

Since government funding is political in nature, political and financial capabilities were particularly intertwined in our data. We expose this further in the next theme.

### 3.2. Financial Capabilities

Financial capabilities are the fit between each tenants’ economic capacity (funds) and the availability of resources to be active in their residential environment. Participants elicited financial capability at the individual, community, and governmental levels.

The cost of physical activity was an important concern to the older public housing tenants we interviewed. They remarked that even simple activities bring on costs. For walking, one must be equipped with good shoes and clothing for the four northern seasons. To swim at public pools, one must have a bathing suit and cap. For some, this was a limiting factor, for others the cost was reasonable. Nevertheless, the potential physical danger was not:

“It doesn’t cost anything, no. The only thing it costs you is your bathing cap, your glasses if you want, but a bathing cap is obligatory, and a bathing suit. You can’t swim naked: you’ll have the pool emptied! […] but I’m afraid to go to the pool and to catch [germs| again. Because there are people like that, like me, who caught germs.”

[B060]

One site had a YMCA in the close neighborhood with a special rate for seniors and people living on a low income. Nevertheless, for all of the participants from this site, it represented too large a portion of their income to invest in: “*To go to the YMCA doesn’t take much, but it’s still $30 per month. That’s some money for us!*” [C030].

If a tenant committee was present, there was a source of collective financial capability. For example, in one site, a former committee president had negotiated with the YMCA nearby to obtain a yoga instructor once a week at much lower costs (C$2 a class per tenant) during the Fall and Winter. The actual president was in the process of renewing this service for which the YMCA covered the remainder of the instructor’s salary. Nevertheless, participants underlined the need for greater financial capability:

“You have to give us volunteers to help us or money, […] If we have money, we can move about. If we don’t have money, we stay put and shut up.”

[C010]

In response to what would be needed to make physical activity more accessible in their residential environment, participants called for financial aid from all of the levels of government. For example, one participant mentioned that a sports association receives municipal funds to subsidize access to a club for individuals living on a low-income. Reduced city public transportation rates for older adults were especially helpful for participants to access physical activity resources within and outside the residential environment. Participants invoked the idea of funding physical activity programs for older adults in public housing, as well as tax breaks for those who stay active. They specified that funding a tenant committee was insufficient, since committees do not stimulate the necessary motivation.

Q: And you spoke about motivation earlier. What can we do to motivate the tenants?

B080: Maybe if the government would send people to these places here, and uh… To get us moving.

Q: To get you moving. What do you imagine? What would you want?

B080: It’s not saying: “Form a committee and do it.” No, no, no. Someone to come and stimulate us. Stimulate us mentally!

Furthermore, tenant committees could be a source of social tension. Indeed, the next theme describes social capability as active.

### 3.3. Social Capabilities

Social capabilities are the fit between the quantity as well as the quality of tenants’ social networks and the opportunities to be active in their residential environment. Participants discussed social capability among tenants in the building as well as among residents in the neighborhood.

Participants described their neighbors in the building as both “family” and “saboteurs”. In one site, the community room was large enough to house physical conditioning equipment (e.g., muscular training stations, stationary bikes, etc.) donated by new tenants. Rather than throw it away, many had chosen to share with others what would not fit in their new dwelling. However, due to the potential for unease or conflict, participants from all of the three sites avoided common spaces in the building. Many participants indicated that they adapted to living in public housing by closing themselves off to neighbors:

“There are activities that many people do with the equipment… There is a pool table for other people who want to play pool too. And another uncomfortable thing is that there are a lot of people that we do not like to see because we know who they are because they are abusers. […] So, it’s the people who make it more or less pleasant for us.”

[A070]

In a different site from that of the previous quote, there was an overt grudge between groups supporting the previous committee or the current committee members. Participants indicated that they would like to contribute to the committee’s organizational activities, but shied away from taking a seat on the committee to avoid the feuds associated with it.

There were examples of informal tenant engagement promoting physical activity in all of the three sites. For example, participants of one site attended physical conditioning sessions that were voluntarily offered thrice a week in the community room by a knowledgeable tenant. Although at the end of data collection, it appeared that these sessions were suspended due to the conflict between the tenants. A tenant in another site facilitated physical conditioning video recordings in the community room until they fell ill. No one took their place, participants said, due to the cliques in the building. To improve social capability for physical activity in the building, some tenants called for a psychosocial intervention, as well as soliciting each tenant’s potential contribution:

“For example, before, when I was president, I saw that every person has a talent. Every person is good at something. And I used their knowledge, I gave them confidence.”

[C060]

Participants living in the mixed area and residential area study sites stated that the neighborhood’s social environment facilitated physical activity. Indeed, they stated feeling safe (more in the daytime) and welcomed by other neighborhood residents. Participants living in the commercial area site mentioned that heavy pedestrian traffic and the regular presence of a boisterous homeless population were a nuisance as well as a safety concern when walking about. Moreover, one participant mentioned that they felt as an outcast in many businesses in the neighborhood, where the median age was much younger given the presence of colleges and universities. Furthermore, fear of crime prevented them from going to places where they did feel more at home, but were unable to carry their walker up or down the stairs:

“I couldn’t go in. […] No because there was a lady in our building who left her walker outside building once and it was stolen.”

[A040]

Community organizations in the surrounding area compensated for the low social capability of being active in the building and neighborhood. Despite having access to training equipment or activities in their buildings, some participants chose to attend community organizations instead. These organizations were specific in scope, tailoring their activities either to older adults or specific cultural groups. The activities were fee-based but considered affordable. Some organizations were beyond the limits of the residential environment. One offered a shuttle service in the icy winter months for an additional weekly cost (C$ 2 dollars). Moreover, these activities promoted active travel for participants commuting to destinations using public transportation. Furthermore, these activities were accessible to participants with relatively good physical capacity. This was not the case for all of the participants, as we explore in the next theme.

### 3.4. Physical Capabilities

Physical capabilities are the fit between tenants’ health and functional status and the opportunities to be active within their residential environment. Participants spoke of physical capability at the individual level, the close residential built environment, as well as the public health promotion tactics conducted in the wider social environment.

Participants expressed that as they aged, they had to accept their declining physical capacity and resign to being less active in their residential environment. One participant who underwent 2 months of hospitalization for an injury stated that they would not use a walker after having worked so hard to regain mobility:

“I tell myself, ‘I have done enough, I’ve done a lot. I’ve done a lot of sports, I’ve done a lot of traveling, going out,’… Like I said, when we, especially in winter, I am a little afraid. Well, it’s scary to fall on the ice! Even if I have a spike on my cane… I won’t take my walker after spending weeks in physiotherapy!”

[B010]

Participants mentioned very few attributes of the built environment that hinder physical activity, which resulted in the interviewer’s interest for why that was the case. They responded that it was not something they ever thought about and that with aging one must accept the situation and adapt their own expectations, since the environment will not change. One participant had expressed their concern of poor access to the community library, without any results:

A040: No ramp. And it’s an old building, they’re not going to spend the money, you know. It’s a big problem here in Montreal. Too many steps, and you have to have for seniors… there’s a ton of seniors using walkers now. Like to enter a building, a ground level is important.

Q: Have you told them?

A040: Oh! Yes! You know they had a suggestion sheet.

Many participants in our sample desired to be more active but felt limited by their knowledge of physical activity. This was especially the case of those with physical incapacity. For example, one participant with an artificial knee and a sensitive hip stated:

“Well, look, a good example, me with the [injured] hip and knee, I don’t know what exercises to do really. Okay? I don’t know. Because I’m thinking to myself, ‘If I put this down and I start doing this, will it bother my hip?’ You know?… I don’t know!”

[B020]

Moreover, participants evoked a need for treatment and self-management support for their medical conditions (e.g., heart disease) or physical incapacity (e.g., hip replacement) in order to be physically active. One participant with a congenital condition causing physical incapacity felt blessed that they could attend specialized and subsidized physical conditioning classes twice a week at a long-term health-care center outside their residential environment. Most of the participants with medical conditions and incapacities did not have access to follow-up or satisfactory information. Possession of knowledge can be considered a psychological resource, which is described in the next theme.

### 3.5. Psychological Capabilities

Psychological capabilities are the fit between tenants’ psychological resources (e.g., knowledge, motivation, resilience) as well as their mental health states and the opportunities for physical activity in their residential environment. Participants described psychological capabilities in relation to their individual psychological resources, namely knowledge and mental health.

Some of the participants stated that the capability to be physically active depended on resources such as access to proper knowledge. For example, one participant was not using the communal physical equipment, since they did not know how to operate it. The written instructions posted on the wall were insufficient for them to feel at ease in using the machines. In contrast, many felt that it was solely their individual responsibility to stay active. For example, in response to “What helps you to move and be active in the neighborhood?”, a participant proclaimed:

“Ah to move and be active it depends on me! I think it depends on me because if I don’t feel like doing something, I wouldn’t do it. If I feel like doing it, [I do it].”

[C090]

Many of the participants suggested that for tenants to be more active a psychosocial intervention would be required to alleviate the distress that tenants experience. Participants suggested that the government subsidize outings to help break up the social isolation they observe among the tenants and one-on-one psychosocial or psychiatric interventions to reduce the emotional depletion described in all of the three sites. One participant eloquently described how the low mental health status of many tenants clashed with their opportunities to be active:

“Because mood here [in public housing] is very important. If you have feelings that weigh on you, on your body, your body is weakened. You don’t even feel like doing these movements. Or to walk. Besides, when you see someone who is demoralized, you tell them to come out and we’ll go out, ‘No, I don’t want to go out’. That happens sometimes. He says no, leave me alone. So, if it repeats itself every time like that, well, the guy gets depressed. He doesn’t want to go out, he doesn’t want to go for a walk. He doesn’t want to because he has, he has another need. That need must be filled first and that need is his feelings that weigh down on the individual.”

[C030]

Rather than complain about what was lacking, they said, many participants expressed gratitude for what they did have, especially those who immigrated from other countries or waited up to 16 years to obtain a dwelling. They were not concerned with walkability in the residential environment since, as one participant puts it, compared to more pressing issues (in this case bedbugs) “…other things just don’t seem really serious”. [A040]. Participants often mentioned a personal responsibility to adapt to their environment, rather than the other way around:

“The unpleasant aspects [of the city] are inevitable and one must accept it. When you get it into your head that it’s normal, it’s not bothersome.”

[A020]

The table below (Table 3) summarizes the results, presenting capabilities for physical activity per socioecological system (first column from the left). It explicitly lists the names of observed existing opportunities (second column) and non-existing opportunities that the participants suggested (third column).

## 4. Discussion

This paper aimed at exploring older public housing tenants’ capabilities to be physically active in their residential environment. Applying a capability approach to analyze 26 walk-along interviews with older public housing tenants, we uncovered five capabilities for physical activity across multiple ecological levels.

Unsurprisingly, financial capability for physical activity was low in all of the three sites. This is consistent with the results of previous studies among general older adults [36,37]. Also, there is a challenging “chicken and the egg” conundrum whereby to feel good a person one should be more physically active, but to be physically active a person needs to feel emotionally well [62,63,64]. The older tenants’ mental health status and its relation to physical activity made up a large proportion of our study’s results in contrast to previous work concerning general older adults’ capability to be physically active [36,37].

Our results are consistent with empirical research showing that older public housing tenants experience more mental health challenges than more affluent older adults [15,17,19,20,21]. Moreover, it is congruent with research among other marginalized populations. Depression was noted as a barrier to physical activity in a study exploring the capabilities of obese and/or diabetic Hispanic adults with a low income in San Antonio (USA) [65]. This congruence between older public housing tenants and adult members of a cultural group living on a low-income suggests a relationship between the greater risk of inactivity and socially determined mental health difficulties among disenfranchised populations. Public housing programs were meant as a social safety net in order for the people with insufficient income to live in dignity [13,49]. Public housing could serve as an outlet to offer tailored services to older adults likely to be living with mental health problems [20]. The results of our study suggest that psychosocial services are needed and are central to physical activity promotion for this subgroup of the population.

Older public housing tenants’ social capability to be physically active related to different social networks compared to studies among general older adults living in Germany. For general older adults, social capability related to family and social support in previous studies [36,37]. Nevertheless, in the current study, older public housing tenants’ social capability to be active was discussed more in relation to friends in the building as well as people encountered in the neighborhood and less in relation to family. This could be due to the fact that our study questions were framed to gather information relevant to the residential environment. Still, other studies have found social capital to be of great influence on health and wellbeing among public housing tenants [66,67]. Improving social cohesion within the building could be a promising avenue to support participation in physical activity.

Older adults’ physical activity depends on changes in mobility and physical capacity in relation to their environment’s ability to meet those changing needs. The built environment currently plays a crucial role in physical activity promotion for older adults [8,13,68,69,70]. The work by Sauter et al. (2019) shows that services in the residential environment did not meet the needs of older adults [36]. Similarly, many participants in our sample chose to travel outside of the close neighborhood to obtain services adapted to their needs. Nevertheless, a few mentioned barriers to physical activity in the neighborhood, in contrast to the barriers to walkability mentioned by the German older adults [36,37], as well as in much other empirical research [8,9,68,69].

Using creative research methods, we learned that rather than highlighting areas for improvement in their environment, some tenants were actively resigning to silence due to a perceived status of low political power. In a commentary discussing older adults’ physical activity, Asiamah (2017) states that the studies that support the activity theory of successful ageing whereby older adults should maintain their activities as much as possible [71] tend to be conducted in more affluent countries with greater individual and collective resources [72]. The studies supporting the disengagement theory whereby older adults naturally and selectively retreat from social roles and life [73] tend to be conducted in less affluent countries [74]. In other words, an older adult’s relation to physical activity may depend on their place in the wider social and political environment, their access to resources, and their perception of control on that environment [72].

It is conceivable that the older adults in Sauter’s (2019) and Frahsa’s (2020) studies perceived greater political capacity and influence on their environments than the participants in our sample, explaining the difference in critique of the built environment between general older adults and older public housing tenants [36,37]. An intersectoral approach that simultaneously considers multiple levels of marginalization (older age, socioeconomic status, cultural identity, gender, etc.) and capabilities for physical activity is warranted to further investigate whether variations in perceived political capacity influence older adults’ capability for and participation in physical activity.

### 4.1. Implications for Practice

Capabilities for physical activity are better understood when considering the interplay of person-environment fit across ecological levels rather than as static or independent factors [44,45]. Indeed, links between various services were fundamental to physical activity capability for older public housing tenants. For example, reduced public transportation rates for senior residents or low-cost organized shuttles allowed the participants to access and attend physical activity services that were adapted to their needs and cultural and ethnic belonging. Since older public housing tenants’ capabilities span across multiple ecological levels and involve multiple stakeholders, physical activity promotion should adopt a concerted action approach such as Health in All Policies with a “Middle-Out” approach [74]. Indeed, our data suggest that “middle-level” actors (e.g., tenant committees and federations, public housing authorities, community organizations, etc.) are crucial to promotion success and that intersectoral capacity building is necessary.

The World Health Organization defines capacity building as a required process to support health promotion by (1) developing practitioners’ knowledge and skills; (2) improving infrastructure across implicated organizations; and (3) developing partnerships and cohesion within the community [75]. The current study supports the previous work showing that capabilities for physical activity are better understood when considering the interplay of person-environment fit across ecological levels rather than as static or independent factors [44,45].

Older adults have the ability to shape their environments and have the right to be given that opportunity [76,77]. The present study has identified different needs and socioecological levels for physical activity promotion among older public housing tenants. As experts of the living conditions in these residential environments, the concerned residents should be included in uncovering successful and acceptable strategies for health promotion [77]. The tenants themselves, the tenant committee, the public housing authority, the community, and the government appear to be ideal stakeholders in promoting physical activity to older public housing tenants. Community-based participatory research methods, such as neighborhood audit walks as well as citizen science methods where concerned members of the community, stakeholders, and researchers evaluate the residential environment on foot then plan and assess change together, have shown promise for physical activity capacity building [78,79,80,81,82,83]. Nevertheless, more evidence on the effects of these initiatives is warranted, given the intricate needs of marginalized populations such as older public housing tenants.

### 4.2. Study Limitations

These findings merit caution for two main reasons. First, we note that this study’s selection criteria pose a recruitment bias to sampling more able individuals. Nevertheless, we included participants with multiple and diverse functional incapacities making participation possible by offering as many breaks as necessary during the walks, by anticipating places where participants could rest en route, and bringing a foldable stool in case resting spaces were far. Second, identifying capabilities in older public housing tenants’ physical activity in their residential environment was not a study objective at inception. Though capabilities were an evident translation theme and best represent our data, we may have identified greater capabilities if the study had been developed with this specific objective in mind. Finally, other capabilities could have emerged if the interviews had been conducted in the cold and icy months of Canadian winter. Though we did inquire about wintertime physical activity, the question was at the beginning of the interview rather than during the walk-along when facilitators and barriers to physical activity were discussed.

## 5. Conclusions

To gain a better understanding of older public housing tenants’ capacities for physical activity in relation to the opportunities to be active in their residential environment, we conducted 26 individual walk-along interviews in three neighborhoods of Montreal (Canada). Thematic analysis produced five themes of capabilities: Political, financial, social, physical, and psychological that span across multiple ecological levels including the individual, the public housing building and its management authority, as well as multiple levels of government. Our results suggest that in order to tailor physical activity promotion to older public housing tenants, efforts should incorporate a psychosocial intervention and adopt an intersectoral partnership approach to work with the tenants from the “Middle-Out” perspective. We contend that using a socioecological capability approach was important in identifying these avenues to foster a more equal opportunity to a fulfilling life for older public housing tenants. Future research should implement and evaluate the outcomes of community-based participatory capacity building methods to foster the wellbeing and physical activity of older public housing tenants.

## Figures and Tables

**Table 1:** Central human functional capabilities [33,34].

Capabilities	Examples
1. Life bodily health	Health conditions (e.g., diabetes, life expectancy)
2. Bodily integrity	Physical ability or disability (e.g., loss of limbs, hip fracture)
3. Senses, imagination, and thought	Ability to take in information (e.g., read, see) learn and reason
4. Emotions	Quality and management of emotional states
5. Affiliation: Living with and toward others	Access to social interactions
6. Affiliation: Having the social bases of self-respect and non-humiliation	Quality of social interactions
7. Practical reason	Ability to discern information and make decisions
8. Political control over one’s environment	Ability to influence power over one’s living conditions
9. Material control over one’s environment	Access to financial or other instrumental means
10. Play	Access and ability to experiences of pleasure and learning
11. Other species	Exposure to and influence of animals and plants on wellbeing

(Adapted from Shinn 2015, p. 245).

**Table 2:** Sociodemographic and perceived health characteristics of 26 older public housing tenants.

Characteristic	N (100%)
Total sample size	26
**Age in years**	
Mean (STD)	71.96 (8.0)
**Sex**	
Women	18 (69%)
**Country of birth**	
Canada	14 (54%)
Other	12 (46%)
**Yearly income**	
C$ 9999 or less	2 (7.7%)
C$ 10,000–19,999	17 (65.4%)
C$20,000–39,999	5 (19.2%)
NRP	2 (7.7%)
**Education level**	
Secondary or less	17 (65.4%)
College diploma	2 (7.7%)
University diploma	7 (26.9%)
Functional comorbidity index	
Mean (STD)	3.40 (2.8)
**Self-reported physical health**	
Very good	11 (42.3%)
Good	10 (38.5%)
Average	4 (15.4%)
Bad	1 (3.9%)
Very bad	-
**Self-reported mental health**	
Very good	6 (23.1%)
Good	11 (42.3%)
Average	7 (26.9%)
Bad	2 (0.8%)
Very bad	-
**Self-reported social support**	
Very good	7 (26.9%)
Good	13 (50.0%)
Average	4 (15.4%)
Bad	1
Very bad	-
NRP	1
**Level of physical activity**	
Low activity	19 (65.4%)
Moderate activity	9 (34.6%)
High activity	0

**Table 3:** Occasions for capability and capacity building per level of the ecological model.

Socioecological System	Existing Opportunities	Suggested by Tenants
Individual	Informal personal engagement in the building and community	Psychosocial intervention Seeking out talents and expertise among tenants
Public housing building and authority	Advocacy/tenant committee Storing equipment in the common room	Psychosocial intervention Offering choice in renovations of common spaces
Community	YMCA Community organizations for older adults and cultural or ethnic groups Not-for-profit sports association	Increased financial support
Government	Municipal funds for cardiovascular equipment (treadmills, elliptical, stationary bike) Sport and leisure services (e.g., Zumba in the park)	Financing health services Subsidizing and planning social outings as well as on-site organized physical activity for older public housing tenants

## Data Availability

The data are not publicly available to protect research participants’ anonymity.
